# Contaminating reactivity of a monoclonal CCAAT/Enhancer Binding Protein β antibody in differentiating myoblasts

**DOI:** 10.1186/s13104-019-4749-3

**Published:** 2019-10-31

**Authors:** Hamood AlSudais, Nadine Wiper-Bergeron

**Affiliations:** 1 Graduate Program in Cellular and Molecular Medicine, Faculty of Medicine, University of Ottawa, 451 Smyth Road, Rm 3106, K1H 8M5, Ottawa, ON Canada; 2 Department of Cellular and Molecular Medicine, Faculty of Medicine, University of Ottawa, 451 Smyth Road, Rm 2539, K1H 8M5, Ottawa, ON Canada

**Keywords:** Myogenesis, C/EBPβ, Myoblast, Antibody cross-reactivity, MYL4

## Abstract

**Objective:**

CCAAT/Enhancer Binding proteins (C/EBPs) are transcription factors involved in the regulation of a variety of cellular processes. We used the Abcam Recombinant Anti-C/EBP beta antibody (E299) to detect C/EBPβ expression during myogenesis. Though the antibody is monoclonal, and the immunogen used is highly specific to C/EBPβ, we identified an intense band at 23 kDa on western blot that did not correspond to any of the known isoforms of C/EBPβ, or family members predicted to cross-react. Absent in myoblast cells overexpressing C/EBPβ, the band was present when C/EBPβ was knocked down, confirming specificity for a protein other than C/EBPβ. The objective of this work was to identify the contaminating reactivity.

**Results:**

We performed immunoprecipitation followed by mass spectrometry to identified myosin light chain 4 (MYL4) as the unknown band, suggesting that the Abcam monoclonal antibody directed against C/EBPβ is not pure, but contains a contaminating antibody against MYL4. Caution should be used when working in cells lines that express MYL4 to not confound the detection of MYL4 with that of C/EBPβ isoforms.

## Introduction

Antibody specificity is key to rigorous and reproducible research findings. Antibodies can be polyclonal, meaning a mixture of antibodies secreted by several clones of B cells in response to an antigen, or monoclonal, where a single clone of B cells is used to produce an antibody with an affinity to a defined epitope. Monoclonal antibodies are known to have high specificity and less background noise, as well as consistency from batch to batch.

Monoclonal antibodies are produced by inoculating mice with a peptide antigen to elicit an immune response. The recovered splenocytes are fused to myeloma cells and then expanded into individual clones to generate hybridomas [1]. All hybridomas thus have a single specificity dictated by the epitope and any cross-reactivity is due to similarity between the inoculating sequence and other proteins [2].

Our laboratory is interested in the regulation of myogenesis by the bzip transcription factor CCAAT/Enhancer Binding Protein beta (C/EBPβ). C/EBPβ is widely expressed and has been shown to play a role in cell differentiation, apoptosis and inflammation [3, 4]. *Cebpb* is an intronless gene that produces three protein isoforms from a single mRNA though leaky ribosomal scanning: Liver-enriched Activator protein* (LAP*), LAP, and LIP (Liver-enriched inhibitory protein) [4–6].

To detect the expression of all protein isoforms of C/EBPβ, antibodies specific to the C-terminus are required. Beginning in 2014, we began validation experiments for a monoclonal anti-C/EBPβ antibody (E299, Abcam, ab32358). Our research focuses on muscle stem cells, called satellite cells, that confer regenerative potential to skeletal muscle [7, 8]. In response to muscle injury, satellite cells become activated, differentiate and fuse to form myofibers that express contractile proteins [8]. In healthy muscle, satellite cells express C/EBPβ which inhibits myogenic differentiation [9, 10]. Upon induction of differentiation, C/EBPβ expression dramatically decreases, allowing differentiation to proceed [9–11]. We report that the anti-C/EBPβ antibody also detects myosin light chain 4 (MYL4) in differentiating myoblasts and in other cell lines. Because MYL4 protein is detected at approximately 23 kDa, this contaminating band can be confused with the LIP isoform of C/EBPβ; therefore, this anti-C/EBPβ should be used with caution in tissues that express MYL4, including skeletal and cardiac muscle.

## Main text

### Methods

#### Cell culture

C2C12 myoblasts (ATCC) were grown in Dulbecco’s Modified Eagle medium (DMEM) with 10% fetal bovine serum (FBS) (GM, growth media) and differentiation was induced by switching confluent cells to DMEM with 2% horse serum (HS). Mouse primary myoblasts were isolated and cultured as previously described [9] and maintained on Matrigel-coated plates in DMEM (Wisent) with 20% FBS (Wisent), 10% HS (Sigma), 10 ng/ml basic fibroblast growth factor and 2 ng/ml hepatocyte growth factor (Peprotech). To induce differentiation, confluent cultures were switched to differentiation media (DMEM, 2% FBS, 10% HS). In vitro Cre recombinase (Cre) fused to a mutant estrogen ligand-binding domain (ER^tm^) (CreER^tm^) activity was induced in primary myoblasts (*Cebpb*^fl/fl^*Pax7*^wt/wt^ (WT) and *Cebpb*^fl/fl^*Pax7* ^CreER/wt^ (conditional knockout, cKO) with 2 µM 4-OH tamoxifen (Sigma) for 48 h. Retroviruses were generated in Phoenix Ampho packaging cells (ATCC) and retroviral transductions performed as previously described [9].

#### Western analysis

Whole cell extracts from primary myoblasts or C2C12 cells were resolved on a 15% SDS-PAGE gel, transferred to Polyvinylidene difluoride (PVDF) membrane (Bio-Rad), and probed with the following primary antibodies: C/EBPβ (E299, ab32358, Abcam), MYL (F5, sc-365243, Santa Cruz), MYL12A/B (A-10, sc-376606, Santa Cruz), MYL4 (ab231800, Abcam) and Cyclophilin B (ab16045, Abcam). The ChemiDoc^TM^ MP system (Bio-Rad) was used to detect chemiluminescence.

#### Immunoprecipitation

Differentiated C2C12 myoblasts were collected in lysis buffer (50 mM Tris-base pH 7.5, 150 mM NaCl, 1% NP-40 and 1X protease inhibitor) and agitated for 20 min at 4 °C. Samples were spun for 10 min at 4000 g and supernatants were collected and precleared with magnetic protein-G-dynabeads (Invitrogen). Approximately 1 mg of protein was incubated with 4 µg of anti-C/EBPβ (ab32358) or non-specific IgG (Invitrogen) for 3 h at 4 °C, while 3% of protein was kept for loading input. Immunoprecipitates were captured using Protein-G-beads and extensively washed in 50 mM Tris-base pH 7.5, 150 mM NaCl and 1% NP-40 and pellets were resuspended in lysis buffer with 5X SDS loading buffer and resolved on a 15% SDS gel for proteomics analysis.

#### Protein Identification by LC-MS/MS

Proteomics analysis was performed at the Ottawa Hospital Research Institute Proteomics Core Facility. Proteins were digested in-gel using trypsin (Promega) according to the method of Shevchenko [13]. Peptide extracts were concentrated by Vacufuge (Eppendorf) and LC-MS/MS was performed using a Dionex Ultimate 3000 RLSC nano HPLC (Thermo Scientific) and Orbitrap Fusion Lumos mass spectrometer (Thermo Scientific). MASCOT software version 2.6 (Matrix Science, UK) was used to infer peptide and protein identities from the mass spectra. The observed spectra were matched against mouse sequences from SwissProt (version 2016-09) and against an in-house database of common contaminants. The results were exported to Scaffold (Proteome Software, USA).

## Results

### Anti-C/EBPβ (E299) antibody from Abcam (ab32358) detects three C/EBPβ isoforms in myoblasts

**Fig. 1:**
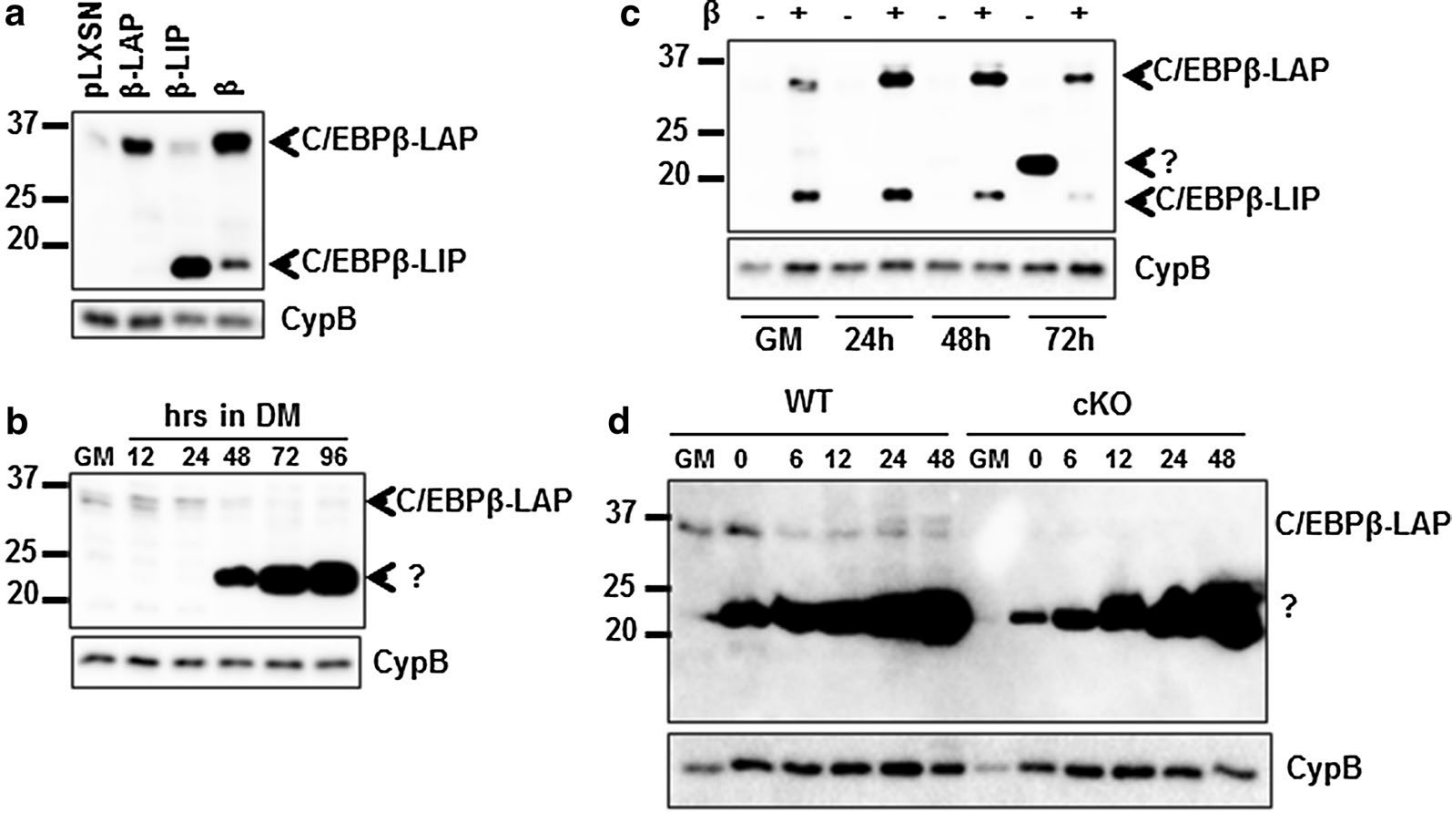
Anti-C/EBPβ antibody E299 detects a ~ 23 kDa band in differentiating myoblasts. **a** C/EBPβ protein expression in proliferating C2C12 myoblasts that were retrovirally transduced to express the LAP isoform of C/EBPβ (β-LAP), the LIP isoform (β-LIP), the full length C/EBPβ (β) or with empty virus (pLXSN). **b** C/EBPβ protein expression in proliferating (growth medium, GM) or differentiating (differentiation medium, DM, 12–96 h) C2C12 myoblasts. Migration of the unexpected band is indicated by “?”. **c** C/EBPβ protein expression in proliferating (GM) or differentiating (24–72 h) C2C12 myoblasts that were retrovirally transduced to express C/EBPβ (β) or with empty virus (pLXSN). **d** C/EBPβ protein expression in proliferating (GM) or differentiating (0–48 h) primary myoblasts isolated from *Cebpb*^fl/fl^*Pax7*^+/+^ (WT) and *Cebpb*^fl/fl^*Pax7* ^CreER/+^ (cKO) mice

To validate the Abcam anti-C/EBPβ antibody in myogenic cultures, we performed western blot of C2C12 myoblasts that were retrovirally transduced to express the LAP isoform (C/EBPβ-LAP), the LIP (C/EBPβ-LIP) isoform, all C/EBPβ isoforms (β) or with empty virus (pLXSN). The antibody successfully detected endogenous LAP, and the LAP (~ 36 kDa) and LIP (~ 17 kDa) isoforms in C/EBPβ-overexpressing cells (Fig. 1a).

### Anti-C/EBPβ antibody (ab32358) detects a non-specific band in differentiating myoblasts

We next evaluated the ability of anti-C/EBPβ to detect endogenous C/EBPβ expression in proliferating and differentiating myoblasts. C/EBPβ expression is highest in proliferating myoblasts, and is rapidly downregulated after induction to differentiate in low serum conditions. C2C12 myoblasts were expanded in growth medium (GM) for 48 h then differentiated in differentiation medium (DM) for 96 h. Consistent with our previous findings [9–11], C/EBPβ-LAP is the most predominantly expressed isoform in myoblasts and its expression decreased 24 h after induction to differentiate (Fig. 1b). However, beginning at 48 h of differentiation, a strong band, far more intense that the signal for C/EBPβ, was detected at ~ 23 kDa and this band was not detected in proliferating myoblasts even with longer exposure time (Fig. 1b). According to the data sheet for the antibody and BLAST analysis of the provided epitope, the antibody could detect C/EBPα and C/EBPε. However, the molecular weight of the observed band does not overlap with C/EBPα (42 and 30 kDa) or C/EBPε isoforms (32, 27 and 14 kDa). To determine if the 23 kDa band represented a novel C/EBPβ isoform, we examined the expression of this band in myoblasts overexpressing C/EBPβ (Fig. 1c). Over the course of differentiation, the band was only detected in empty vector control cells at 72 h of differentiation, while it remained undetectable in cells overexpressing C/EBPβ (Fig. 1c). We next examined the expression of this band in primary myoblasts isolated from a conditional knockout mouse (cKO) in which *Cebpb* is excised in satellite cells. Satellite cells were cultured in growth medium for 48 h and switched to differentiation medium for 48 h (the time course for differentiation of primary myoblasts is shorter than in C2C12 cells). Knockout efficiency was confirmed by western blot (Fig. 1d) and C/EBPβ-LAP expression in WT cells was downregulated with differentiation as previously reported [9, 10] (Fig. 1d). Interestingly, the 23 kDa band was detected in differentiating WT and cKO myoblasts ruling out the possibility that this band is an isoform of C/EBPβ (Fig. 1d). Since C/EBPβ is an inhibitor of myogenesis [9, 10], the detection of the 23 kDa band correlates with myogenic differentiation (detected only in differentiating myoblasts) and not with C/EBPβ expression (Fig. 1c, d).

### Anti-C/EBPβ detects MYL4 in differentiating myoblasts

**Fig. 2:**
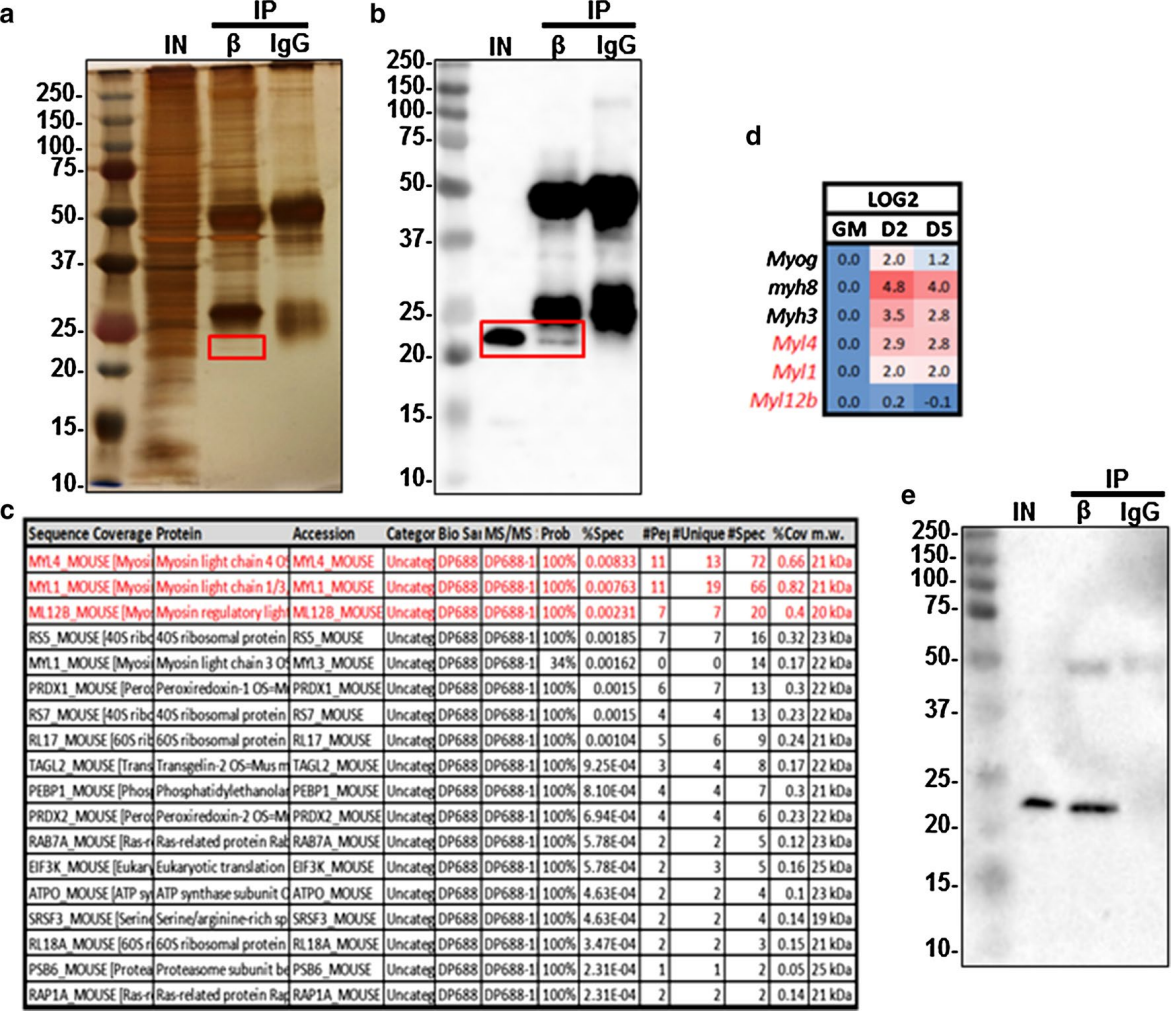
LC-MS/MS analysis of the 23 kDa band identifies 16 mouse proteins. **a** Migration of proteins immunoprecipitated by anti-C/EBPβ antibody (ab32358) or non-specific rabbit IgG from differentiating C2C12 myoblasts, stained with silver stain. Input is 3% of material used for immunoprecipitation. The rectangle highlights the bands that were subject to MS analysis. **b** Western blot analysis of the anti**-**C/EBPβ IP samples performed as in **a** with a rectangle identifying the unknown band. **c** Scaffold Viewer data for mouse proteins detected by MS from **a**. **d** *Myog,Myh8, Myh3, Myl4, Myl1* and *Myl12b* gene expression in proliferating (GM) primary myoblasts and differentiated for 2 and 5 Days (D2 and D5) obtained from publicly available microarray data (GSE24811) [12]. Data are presented as Log2 to the GM samples. **e** Western blot analysis of the anti**-**C/EBPβ IP samples performed as in **a** and incubated with anti-MYL4 (ab231800)

To identify the protein causing the 23 kDa band in differentiating myoblasts, we performed an immunoprecipitation (IP) of whole cell extracts from C2C12 myoblasts differentiated for three days using the anti-C/EBPβ antibody or non-specific IgG. The 23 kDa band was successfully precipitated using the anti-C/EBPβ antibody but not by the control IgG as detected by silver staining (Fig. 2a, red box). Western blot analysis of the input and the C/EBPβ-IP sample confirmed the pull down of the 23 kDa band, and its absence in the control IP lane (Fig. 2b). The excised 23 kDa band was analyzed by mass spectrometry, which identified 16 mouse proteins with molecular weights between 19 and 23 kDa (Fig. 2c). Based on the spectrum counts, myosin light chain proteins (MYL4, MYL1/3 and MYL12b) were detected at higher levels than others. Similarly, myosin light chain proteins were more highly ranked based on the percentage of amino acids detected by the spectrum. Myosin light chain 4 was detected with 11 exclusive unique peptides, 13 exclusive unique spectra and 72 total spectra with 66% coverage (Fig. 2c). Myosin light chain 1/3 skeletal muscle isoform (MYL1) was detected with 11 unique peptides, 19 unique spectra, 66 total spectra and 82% coverage. MYL12b was also identified with 7 unique peptides and 40% sequence coverage. We used publicly available microarray data from proliferating (GM) and differentiated myoblasts (GSE24811) [12] to determine the expression pattern of the myosin light chain genes. In parallel with myogenin and myosin heavy chain 8 and 3 (markers of terminal differentiation), *Myl4* and *Myl1* were upregulated with myogenic differentiation (Fig. 2d). *Myl12b* gene expression remained stable with differentiation (Fig. 2d).

**Fig. 3:**
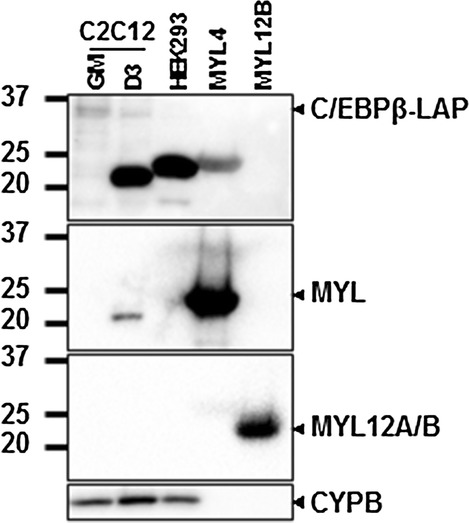
Anti-C/EBPβ antibody (ab32358) detects MYL4 in differentiating myoblasts. Western blot of extracts from proliferating myoblasts (GM), 3-day differentiated myoblasts (D3) and HEK293 cells compared to human recombinant MYL4 (ab115722) and human recombinant MYL12B (ab128438) probed with anti-C/EBPβ (ab32358), anti-MYL (sc-365243) and anti-MYL12A/B (sc-376606). Cyclophilin B is used as loading control

To confirm the identity of the band detected with the anti-C/EBPβ antibody, we incubated the membrane from Fig. 2b with antibody against MYL4, confirming the detection of MYL4 protein in the C/EBPβ-IP sample at a similar level to the input (Fig. 2e). Furthermore, a western blot comparing extracts from undifferentiated myoblasts (GM), differentiated myotubes (D3), extracts from HEK293 cells (human) and recombinant myosin light chain proteins was performed (Fig. 3). The anti-C/EBPβ antibody failed to detect recombinant MYL12B, but did recognize recombinant MYL4 protein, as well as a band in HEK293 cells (Fig. 3). Interestingly, while HEK293 cells are not thought to express MYL4, human MYL4 is known to be slightly larger than the mouse protein. Loading of recombinant proteins was verified using an antibody that detects multiple isoforms of MYL (MYL1, MYL3, MYL4 and MYL6), which also detected a band in differentiated C2C12 extracts but not HEK293 cells, or against MYL12A/B. The anti-C/EBPβ antibody failed to recognize recombinant MYL1 (data not shown). Thus, these findings suggest that the previously unidentified band detected by anti-C/EBPβ in differentiating myoblasts corresponds to MYL4.

## Discussion

The advancement of scientific knowledge relies on the capacity to build on shared knowledge. At the heart of this is the choice of tools, including antibodies that are validated both by suppliers and researchers. Herein, we describe the validation of an anti-C/EBPβ antibody that is reported to detect all three C/EBPβ protein isoforms, but also shows specificity for MYL4, which is present in differentiating myoblasts, myotubes and myofibers, and numerous other cell lines, including commonly used cancer cell lines and cardiac muscle. Because the molecular weight of MYL4 is close to that of the C/EBPβ-LIP isoform, in tissues and cells that also express MYL4, of which many do, caution must be used when identifying the LIP isoform, and researchers should use extreme caution when using this antibody to detect C/EBPβ by immunofluorescence.

Interestingly, the anti-C/EBPβ antibody also detected a band in HEK293 cells, which was not detected by antibodies that recognize MYL1/3/4/6 proteins. While HEK293 cells do not express these proteins, there is high sequence conservation between MYL family genes. As such, the band detected in the HEK293 cells using the anti-C/EBPβ antibody may represent cross-reactivity with a non-muscle human isoform of MYL that has sequence identity with mouse MYL4.

The anti-C/EBPβ antibody is described as a monoclonal antibody. However, the presence of at least two specificities in the absence of similarity between the epitope and the MYL4 sequence suggests that the hybridoma used to produce this antibody is the most likely source of the contamination, with the presence of at least 2 clones of antibody-producing cells, one for anti-C/EBPβ antibody and one that produces the anti-MYL4 antibody.

## Limitations

In our mass spectrometry experiment, we did not isolate and analyze a gel slice corresponding to the migration of the C/EBPβ-LAP isoform, which would have further confirmed the dual specificity for the antibody.

## Data Availability

All data generated or analyzed during this study are included in this published article.

## Abbreviations

C/EBP: CCAAT/Enhancer Binding Protein
MYL: myosin light chain
LAP: liver-enriched activating protein
LIP: liver-enriched inhibitory protein
DMEM: dulbecco’s modified eagle medium
FBS: fetal bovine serum
HS: horse serum
GM: growth medium
DM: differentiation medium
SDS-PAGE: sodium dodecyl sulphate–polyacrylamide gel electrophoresis
PVDF: polyvinylidene difluoride
LC-MS/MS: liquid chromatography with mass spectrometry
HPLC: high performance liquid chromatography
